# Prevalence and Outcome of Snake Bites Among Children Admitted in the Emergency Pediatric Unit, Federal Medical Centre, Birnin Kebbi, Nigeria

**DOI:** 10.7759/cureus.17413

**Published:** 2021-08-24

**Authors:** Usman A Sanni, Taslim O Lawal, Tawakaltu L Musa, Abdurrazzaq Alege, Aliyu M Na'uzo

**Affiliations:** 1 Paediatrics, Federal Medical Centre, Birnin Kebbi, NGA; 2 Paediatrics, Federal Medical Centre, Katsina, NGA

**Keywords:** snakebite, child, prevalence, anti-snake venin, signing against medical advice (sama), absconded, outcome, birnin kebbi, nigeria

## Abstract

Introduction

Snakebites are common and constitute an important health problem in many countries of the world, with the greatest burden occurring in rural areas of Asia and Sub-Saharan Africa. They were classified by the World Health Organization as category A of neglected tropical diseases. Most studies on snake envenoming in Nigeria were among adult populations with few among children. This study assessed the prevalence and outcome of snakebite among children in Federal Medical Centre, Birnin Kebbi.

Methods

This was a four-year retrospective study in which the medical records of patients with managed snakebite were reviewed. A study proforma was used to obtain information on socio-demographic characteristics, site of the bite, features of envenoming, pre-hospitalization intervention, hospital treatment, length of hospitalization, and outcome of treatment of the patients.

Results

There were 19 snakebite cases out of 5,195 admissions during the period under review, giving a prevalence of 0.0037 (3.7/1000) with a male:female ratio of 2:1. The majority (66.7%) of the children were aged between 11 and 15 years and the mean (± SD) age of the study population was 10.5 (± 3.3) years. The lower limb was the site of bite in 10 (55.6%) of the patients and clinical features included local pain (100%), local swelling of varying magnitude (16 (88.9%)), spontaneous bleeding eight (44.4%) among others. Ten (55.65%) patients presented after four hours of bite and the mean (±SD) duration of hospitalization was 2.11 (±0.58) days. Most (77.8%) received at least one form of pre-hospital care while only 66.7% received polyvalent anti-snake venin. The case fatality rate was 5.6% while 55.6% of patients signed against medical advice.

Conclusion

There was a low hospital prevalence of snakebite in children in the present study location with associated low mortality but a high rate of discharge against medical advice. Most of the patients had a pre-hospital intervention and anti-snake venin is not readily accessible.

## Introduction

Snakes are widely distributed globally though with certain exceptions in frozen environments and high altitudes like the Arctic, Antarctic, and many small islands [1-2]. Snakebites are common and constitute an important health problem in many countries of the world. The greatest burden occurs in rural areas of Asia and Sub-Saharan Africa where snakes are abundant and human activities largely agro-based. In Sub-Saharan Africa (SSA), at least 500,000 snakebite envenoming occur annually, resulting in about 30,000 deaths with a similar figure of definitive disabilities [3]. This represents more than 20% of all notified snakebites envenoming worldwide [3]. In Nigeria, snake bites peak at times of the early rainy season and harvesting periods; mainly in rural areas with limited access to prompt and effective treatment [4]. An earlier study on snake bites in the Northern region of Nigeria put the annual incidence at 497/100,000 with a mortality rate of 12.2% [3,5]. In 2017, the World Health Organization (WHO) added snakebite envenoming to category A of neglected tropical diseases (NTDs), and the WHO Snakebite Envenoming Working Group (WHO-SBEWG) was created [5]. In May 2019, WHO also launched a program to prevent and control snakebite incidents through improved access to effective and safe treatment for the communities most affected, with a target to reduce snakebite mortality and morbidity by 50% by 2030 [6].

There are various types of snakes. However, in the West African sub-region, including Nigeria, those considered medically important include Echis carinatus (saw-scaled carpet viper), Naja nigricollis (spitting cobra), and Bitis arietans (puff adder) [7]. These species account for most of the mortality and morbidity associated with snake bites in the sub-region [7].

Though an earlier study on snakebite was carried out across northern Nigeria, including the present study location [8], the study focused on the knowledge of the health workers on the treatment of snake bites and not the patients managed. Also, a number of earlier studies on snake envenoming within Northern Nigeria were among adult populations with very few among the pediatric age group. Meanwhile, children tend to sustain more severe toxicity from envenoming because of an increased venom: body mass ratio compared to adults [9-10]. Hence, the manifestations and outcomes in pediatric and adult populations may not be the same. Consequently, it is essential to investigate snakebites among pediatric age-group in different localities. This study, therefore, sought to determine the prevalence, presentation, and outcome of snake bites among children admitted to Federal Medical Centre, Birnin Kebbi, Nigeria.

## Materials and methods

Study setting/location

This study was carried out at the emergency pediatric unit (EPU) of Federal Medical Centre (FMC), Birnin Kebbi. It is the referral federal tertiary health institution in the entire state located in North-Western Nigeria.

Study design

The study was retrospective, covering a period of four years (July 1, 2017, to June 30, 2021).

Sample size

All children, aged three to 14 years, managed for snake bites at the EPU of FMC, Birnin Kebbi, during the study period were included.

Ethical consideration

Ethical approval for the study was obtained from the Federal Medical Centre research ethics review committee.

Data collection

The case records of all snake bites were retrieved from the hospital medical record department. Information extracted and entered into a predesigned study proforma data sheet included age, gender, geographic location of the bite, region of the patients’ body bitten, features of envenoming, treatment given before and during hospitalization, use of polyvalent anti-snake venom (ASV), length of hospital stay, and outcome of treatment. One patient whose case record could not be traced from the medical record department was excluded from the study.

Data were entered into Microsoft Excel 2016 version (Microsoft Corporation, Redmond, NY) and analyzed. Categorical variables were summarized using proportions, pie charts, and bar charts, whereas means and frequency tables were used to illustrate quantitative data.

## Results

During the period under review, there were a total of 5,195 pediatric admissions to the emergency pediatrics unit (EPU) of Federal Medical Centre, Birnin Kebbi. Of these, 19 were cases of snakebite, giving a prevalence of 0.0037 (3.7/1000). However, the case note of one patient couldn’t be traced.

There were 12 males and six females with a male:female ratio of 2:1, and most (66.7%) of the children were from semi-urban areas. The majority (66.7%) of the children were aged between 11 and 15 years and the mean (± SD) age of the study population was 10.5 (± 3.3) years.

As shown in Table 1, the geographic locations of the bite have equal distribution, with 50% of all the patients either bitten at home or on the farm The geographic location of the bite in relation to gender is however different; 83.3% of the female subjects were bitten at home while 66.7% of the males were bitten on the farm. Of the nine cases bitten at home, the bite took place while walking and/or playing around the house premises in seven patients and during sleep outside the house in two patients. Bites on the farmland occurred while victims were assisting their families on the farm or while collecting firewood. One child was bitten on the face while attempting to carry foliage on the head within which was the snake. More than half (55.6%) of the snakebites occurred in the evening/night.

**Table 1:** Demographic characteristics of the patients

Variable	Male n(%)	Female n(%)	Total (%)
Age			
0-5	0 (0.0)	2 (33.3)	2 (11.1)
6-10	1 (8.3)	3(50.0)	4 (22.2)
11-15	11(91.7)	1(16.7)	12 (66.7)
Total	12(100.0)	6 (100.0)	18 (100.0)
Geographic location			
Semi-urban	9 (75.0)	3 (50.0)	12 (66.7)
Rural	3 (25.0)	3 (50.0)	6 (33.3)
Place of bite			
Home	4 (33.3)	5 (83.3)	9 (50.0)
Farm	8 (66.7)	1 (16.7)	9 (50.0)

**Figure 1:**
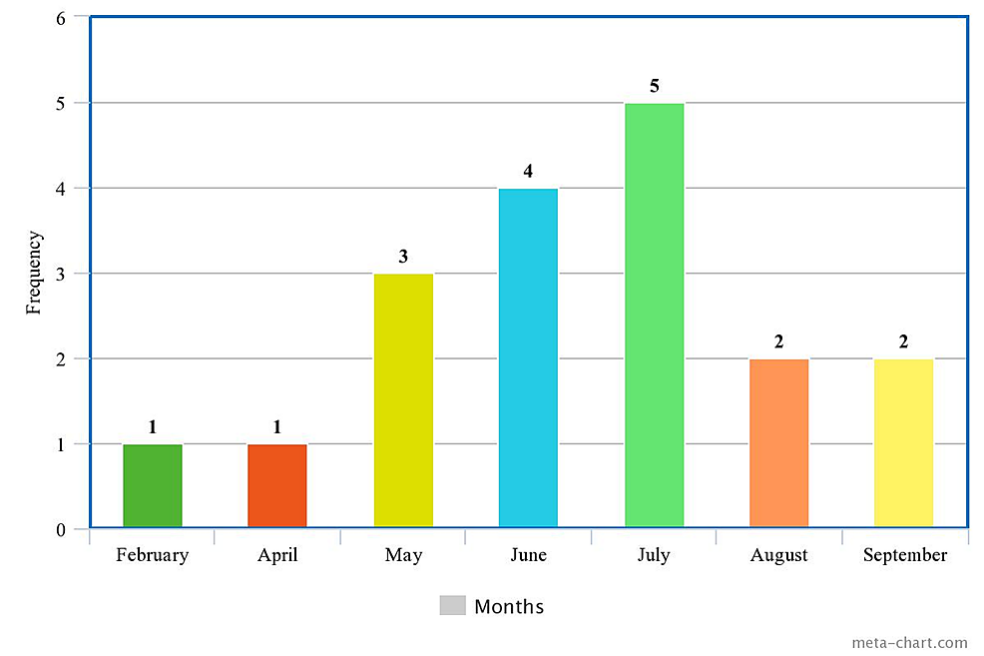
Distribution of snakebites cases by month

Figure 1 shows the monthly distribution of snakebites in the study area. Most of the snakebites occurred between May and September.

As illustrated in Table 2, the lower limb was the site of the bite in 10 (55.6%) of the patients while two (11.2%) had a bite to the head region (one (5.6%) to the face while sleeping within the home environment and one (5.6%) while carrying foliage on the head within which was the snake).

**Table 2:** Clinical features, site of bite, complications, interval between bite and presentation, duration of hospitalization ¥: Multiple responses possible

Variable	N (%)
Clinical features ¥	
Pain	18 (100.0)
Swelling	16 (88.9)
Spontaneous bleeding	8 (44.4)
Vomiting	5(27.8)
Lethargy	5 (27.8)
Dizziness	3 (16.7)
None	2 (11.1 )
Site of bite	
Lower limb	10 (55.6)
Upper limb	6 (33.3)
Forehead	1 (5.6)
Face	1 (5.6)
Total	18 (100.0)
Complication	
Ulcer	6 (33.3)
Bullae	5 (27.8)
Compartment syndrome	3 (16.7)
Interval between bite and presentation	
≤ 4 hours	8 (44.4)
>4 hours	10 (55.6)
Duration of hospitalization	
<1 day	2 (11.1)
1-5	12 (66.7)
6-10	4 (22.2)

The type of snakes involved was identified by proxy using verbal description/ local names of the snakes by patients’ relatives in 12 (67%) patients, including one of a patient’s relatives that came to the hospital with the killed snake, as a cobra in two and a carpet viper in 10 cases.

The clinical manifestations/sign of envenoming are shown in Table 2. All (100%) of the victims presented with pain while 16 (88.9%) had local swelling of varying magnitude. Prolonged clotting time (>20 min) occurred in 10 (61.1%), 8 (44.4%) of whom had spontaneous bleeding. Two (11.2%) of the patients had no signs of envenoming other than local pain. Three (16.7%) patients developed compartment syndrome.

The majority (55.65%) of the patients presented after four hours following a bite and the duration of hospitalization was one to five days in 12 (66.7%) of the patients with a mean (±SD) duration of 2.11 (±0.58) days among the patients.

Pre-hospital treatment and hospital care received by the patients are illustrated in Table 3. Only four (22.4%) of the patients received no form of treatment prior to presentation to the hospital while the majority (14; 77.8%) received at least one form of unorthodox interventions/first aid treatment from traditionalists in the forms of herbal concoctions (topical/oral), local incisions, and prolonged application of a tourniquet to the affected limb.

**Table 3:** Treatments received ASV: polyvalent anti-snake venom ¥: Multiple responses possible

Treatment ¥	N (%)
Pre-hospital treatment ¥	
Tourniquet	9 (50.0)
Topical herb	9 (50.0)
Oral herb	7 (38.9)
Incision	3 (16.7)
None	4 (22.2)
Hospital care ¥	
Analgesics	18 (100.0)
Tetanus toxoid	18 (100.0)
Antibiotics	15 (83.3)
ASV	12 (66.7)
Blood transfusion	4 (22.2)

All patients were administered analgesic and tetanus toxoid. Polyvalent anti-snake venin (ASV) was prescribed for all the 16 (88.9%) patients with signs of envenoming but was procured and administered in 12 (66.7%) with only five (39%) having the prescribed dose. None of the patients developed an adverse reaction to the ASV nor a hypersensitivity reaction to the test dose. Four patients had anemia requiring blood transfusion.

**Figure 2:**
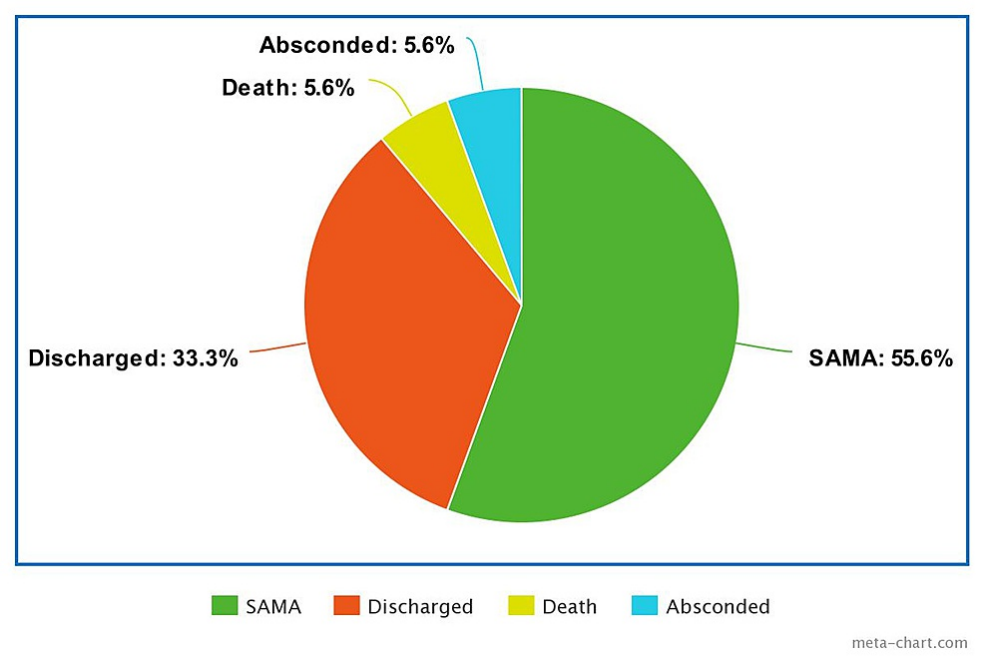
Outcome of hospital treatment SAMA: signed against medical advice

The outcome of management of cases of snakebite is depicted in Figure 2. The majority (55.5%) of the patients left against medical advice and the case fatality rate was 5.6%.

## Discussion

The low hospital prevalence of snakebite among children reported in this study was similar to the findings in Sokoto, another North Western state, as well as Enugu, South Eastern Nigeria [4,11-12]. The reported prevalence in the present study, most likely, is an underestimation of the burden of the problem in the study location. This was in contrast to a high prevalence of snakebite earlier reported in sub-Saharan Africa [3,13]. The low hospital prevalence of snakebite may be common to many resource-limited countries where health-seeking behavior, health beliefs, and access to health care are suboptimal [14]. Hence, it may be that many victims sought care from the traditional/unorthodox healers because of these aforementioned reasons. It may also be that quite a number of them died at home prior to getting to the hospital. These postulations can further be corroborated by community-based studies from rural Nigeria and Kenya that estimated that only 8.5% and 27% of snakebite victims, respectively, sought hospital treatment [15-16]. Furthermore, a rural Bangladesh study revealed that only 3% of snakebite cases presented directly to a health care facility [17]. Contrary to these findings, some hospital-based studies carried out in resource-limited settings had larger study populations [18]. These studies had certain peculiarities that favored the large study populations. For instance, a study in India had a large sample size, partly because it was carried out in a rural setting where ASV was also made readily accessible [19]. Furthermore, the snakebite study carried out among children in Eastern Nepal had a large sample size of 395 [20]. This might be due to the fact that it was carried out at a snakebite-dedicated facility where ASV was also provided to patients requiring it thereby increasing access to ASV at no cost. These observations in low resource countries are, however, not in tandem with the experience in high-income countries with though a low prevalence of snakebite but more accurate data, perhaps due to a broader and more equitable distribution of health services even in their rural communities [14].

The pattern of distribution of patients in this study was similar to reports from earlier studies. Snake bites were more common between May and July, which coincides with the onset of the rainy season with intense farming activities. Also, during this period, the holes and burrows occupied by snakes and rats are filled with water thus rendering the snakes with no shelter [18]. These possibly resulted in increased exposure between man and snake, resulting in more cases of snakebites in humans during the period. The majority of the children managed were males similar to the findings from Sokoto (Nigeria) [4], rural India [19], Eastern Nepal [20], Sri Lanka [13], and Arizona, United States of America [10]. The higher prevalence among male adolescents could stem from their more adventurous nature in addition to participating in such activities as agricultural practices and firewood collection, along with adults, which may predispose to snake bite as noted by earlier studies [4,19-20]. The cultural and religious practices in the study location, similar to Sokoto [4], which encourages females to stay more indoors thereby reducing their vulnerability to snake bites particularly from outdoor farming activities, could account for the lower proportion of females in the present study. The majority of the female subjects in the present study were bitten at home, further buttressing the earlier statement. This, however, contrasted with the report from an Enugu study [11] with more female preponderance and a Costa Rican study [21] with no gender difference. Reasons for those observations were not stated in the aforementioned studies.

Furthermore, most cases in our study came from semi-urban areas located close to the study site. It may be because those semi-urban dwellers are better informed. They are, therefore, more likely to seek hospital care as against patronizing traditional healers. The occurrence of snakebites around the homes, sometimes at night, may underscore possible poor environmental conditions, including poor illumination around the homes. This poor environmental condition could have offered habitats to the snakes close to human dwellings. Furthermore, the poor illumination around the homes in the night might have prevented the detection of the snakes, some of which may have nocturnal activity. These possibly resulted in those bites in humans. This calls for adequate environmental sanitation and maintenance.

The majority of our patients developed signs of envenoming; only two of the patients had no such sign. This indicates that the majority of the bites are from venomous snakes even though the snakes were not identified in some instances. The most common presenting features among our patients included local pain and swelling of variable severity, as well as bleeding. This was similar to findings in other studies in the tropics [4,11]. This perhaps suggests similarity in the distribution of the snake species. Also, the lower extremity was the commonest part of the body involved in most bites in the current study. This was in tandem with findings by other researchers [11,13,19-20]. This could be due to accidental stepping on the snakes. It was earlier reported that in the tropics, snakebites occur more on lower extremities as victims were usually bitten while treading upon or near the snakes [22]. On the other hand, most bites in non-tropical countries occurred more on the fingers and hands following deliberate contact with the reptiles [22]. Bites in the head/neck region, with risk of early systemic envenoming and possible poor outcome (being closer to the heart and the central nervous system), were earlier reported by Belonwu et al. in a Nigerian child [23]. This was the least affected part of the body in our study, found in only 11% of the patients. This was similar to 7% involvement in a Sri Lankan study as well as findings from other Nigerian studies [4,12-13]. It may be that the said region of the body is far from easy reach for the snakes.

Quite a number of patients in the present study had pre-hospital care. This was in form of herbal medications (both oral and topical), local incision, and prolonged tourniquet application. These cultural practices widely practiced in our environment are contrary to the recommended WHO pre-hospital care for patients with snakebite [5]. These practices have also been shown to be unhelpful and may even worsen the patient’s condition and outcome in some instances [24-25]. It might also lead to a delay in presentation to the health facility, as seen in the majority of our patients, thereby militating against the administration of snake antivenin within four hours of the bite as recommended by the WHO [26].

Hospital care of the patients included administration of polyvalent anti-snake venin (ASV), antibiotics, tetanus toxoid, and in few cases, blood transfusion. ASV was prescribed in all the patients with envenoming. However, only 66.7% of the patients procured and had it administered, though not all procured the required doses. This may be a result of the high cost of the antivenin thereby limiting its universal access. None of our patients developed a skin reaction to the test doses prior to administration of ASV. This was in contrast to an American study [10]. Although in the American study, the seven children who had positive skin tests prior to ASV administration or were known to be allergic to horse serum were pre-medicated with epinephrine, histamine blockers, or steroids prior to receiving ASV, and all tolerated the infusion without immediate hypersensitivity. Skin testing and prophylactic premedication of every child presenting with suspected snakebite, however, still remain controversial [1,27]. Furthermore, none of our patients developed an allergic reaction to the ASV contrary to 3% to 50% reported in previous similar studies [4,10-11]. The observed difference could be premised on the differences in the types and constituents of the antivenins. It might, perhaps, also be due to the differences in the genetic makeup of the subjects. In addition, it might also be because a number of our patients did not get the required number of doses (vials) of the antivenin due to financial constraints.

The case fatality in this study was low. This was in consonance with findings in similar studies from different parts of Nigeria and other tropical areas [4,11-12,24]. Furthermore, a number of hospital studies globally have also demonstrated that a snake bite is most often associated with low mortality. This may be corroborating the postulation of researchers that bites from venomous snakes are frequently ‘dry bites’ with low volume or no envenoming, resulting in low case fatality [28]. This could further be illustrated by a study conducted at the Toxinology and Toxicology Unit of the General Hospital of the Central Province of Peradeniya, Sri Lanka, which demonstrated that up to 86% of the 776 snakebite admissions, received a bite in which no venom was injected [29]. On the other hand, the reported mortality rates in the aforementioned hospital-based tropical studies could be underestimations. This statement can be substantiated with the finding in a study in Monaragala District of Sri Lanka in which data on snakebite mortality in all hospitals in the district were compared to data on snakebite as the certified cause of death for the district, for a five-year period (between 1999 and 2003). It was discovered that hospital statistics did not report 62.5% of the true number of snakebite deaths in the district [30]. Furthermore, the high rate of signing against medical advice (SAMA) among patients in the current study was an important factor to be seriously considered before concluding on low case fatality. This is because the final outcome (survival/demise) could not be ascertained, as those patients were lost to follow-up, and caregivers were not contacted subsequently. Therefore, the actual mortality rate among the patients may be higher than the documented hospital figure.

## Conclusions

There is a low facility prevalence of snakebite in children in the present study location with an associated high rate of discharge against medical advice. Most patients presented late and had received at least one form of pre-hospital care. ASV is not readily accessible in the study location.

There is a need for a community-based cross-sectional study to ascertain the actual burden of snake bites among children in our locality. The government should make ASV available at no or a subsidized price to patients in addition to strengthening the health insurance scheme. There is also a need for creating increased public awareness on the effective management of snakebite in children.
